# Epidemiological analysis of congenital glaucoma: a national scenario

**DOI:** 10.1590/1806-9282.20231203

**Published:** 2024-05-03

**Authors:** João Pedro Gambetta Polay, Fabio Vinicius Barth, Renata Nadal Bayer, Camila Ost

**Affiliations:** 1 Universidade Estadual de Ponta Grossa, Department of Medicine – Ponta Grossa (PR), Brazil.; 2 Universidade Estadual de Ponta Grossa, Maternal and Infant University Hospital – Ponta Grossa (PR), Brazil.

**Keywords:** Glaucoma, Epidemiology, Infant, newborn, diseases

## Abstract

**INTRODUCTION:**

Congenital glaucoma is a disease that involves increased intraocular pressure and can result in irreversible visual deterioration. The study of epidemiology allows the delineation of the characteristics associated with patients and specific risk factors.

**OBJECTIVE:**

The objective of this study was to examine epidemiological trends, place of residence, duration of gestation, sex, and race of the newborn diagnosed with congenital glaucoma in Brazil.

**METHODS:**

Data from SINASC (National Live Birth System) were used to analyze the period from 2017 to 2021 in Brazil. Linear regression and analysis of variance tests were employed to assess significance. The statistical significance was determined by p<0.05.

**RESULTS:**

A total of 47 cases of congenital glaucoma were identified in Brazil during the study period, with the highest incidence between the years of 2018 and 2021. The analysis of the distribution indicated that the states with the highest incidence were São Paulo, followed by Rio Grande do Sul and Pernambuco. Approximately 60% of cases occurred in male individuals, compared with 19 female cases. The ethnic analysis showed the highest incidence among whites and mixed. Regarding the length of pregnancy, statistical differences were observed between newborns of different periods of gestation. Infants born from pregnancies lasting between 28 and 31 weeks and 32 and 36 weeks were significant when analyzed with the group between 37 and 41 weeks.

**CONCLUSION:**

Studies on the mechanisms of congenital glaucoma seek to improve knowledge about the disease. Epidemiological evaluation is essential for identifying demographic and clinical patterns of the disease.

## INTRODUCTION

Glaucoma is a group of conditions that are defined by the presence of optical neuropathy, which has the potential to progressively deteriorate over time. This condition is closely associated with an elevation in intraocular pressure (IOP), which can initiate an irreversible process leading to visual degeneration^1^. As a result of the prevalence of hereditary disorders within the category of pathologies that contribute to juvenile vision loss, congenital glaucoma is identified as a relatively uncommon condition, accounting for around 20% of childhood blindness diagnoses^2,3^.

In the field of epidemiology, it has been discovered that congenital glaucoma occurs in around 1 out of every 10,000 persons, with a higher prevalence among males^4-6^. The pathology's underlying etiology is connected with an aberration in the drainage mechanism of the aqueous humor. This defect is known as primary congenital glaucoma, and it can also be secondary to other clinical or iatrogenic disorders. As a consequence, it leads to increased IOP^7^.

The majority of cases of congenital glaucoma are considered random, while around 10% of cases have a recessive genetic pattern. The physiopathology of this condition involves a disruption in the development of the trabecular meshwork architecture and the angular shape of the anterior chamber^7^. However, there is no apparent association with other significant ocular abnormalities. The condition known as isolated trabecular dysgenesis is distinguished by the absence of a specific portion of the ciliary body, resulting from the occlusion of the trabecule by a transparent amorphous material^4,7^.

The patient commonly exhibits symptoms such as excessive tearing, involuntary eyelid spasms, and sensitivity to light. Furthermore, manifestations such as corneal edema and opacity, excavation of the optical disk, and abnormal expansion in the size of the eyeball can also be observed. The clinical assessment necessitates the performance of many tests, including ophthalmoscopy, tonometry, gonioscopy, and pachymetry. These procedures are crucial for both the diagnosis and monitoring of this particular condition^4,8-10^.

The diagnostic criteria encompass a positive IOP measurement over 21 mmHg, accompanied by certain structural alterations, such as a corneal diameter increase of more than 1.5 mm or an asymmetry of more than 1.5 mm. Furthermore, these characteristics encompass the concomitant occurrence of progressive myopia, accompanied by an augmentation in the corneal diameter and/or axial length of the ocular organ, an escalated excavation of the optical disk exceeding 20%, and the necessity for surgical intervention to regulate IOP^11,12^.

Surgical operations are frequently employed as the primary therapeutic strategy, which has the potential to maintain visual function for an extended period of time^13^. The initial procedure often involves the performance of goniotomy, although, in certain circumstances, further interventions such as trabeculectomy and trabeculotomy may be necessary^2,4,14^.

The formation of a diagnosis for congenital glaucoma is heavily reliant on the identification of the observed pattern in afflicted people, given the symptoms associated with this condition are non-specific^15^. In this particular context, it is imperative to undertake a comprehensive national epidemiological study on this particular pathology. This study acts as a fundamental approach to delineate the characteristics linked to patients, with the ultimate goal of estimating specific risk factors and enhancing diagnostic considerations within the field of pediatric ophthalmology.

## METHODS

This study encompasses a quantitative research methodology, which is distinguished by its descriptive approach and utilization of a cross-design. The data collection process for recording cases of congenital glaucoma (CID 10 Q150) involved utilizing the information provided in the "Anomalia ou defeitos congênitos em Nascidos Vivos" section inside the SINASC database, which is accessible through the DATASUS portal^16^. This platform covers the entirety of the Brazilian area. The data set encompasses a period between 2017 and 2021. The variables examined in this study comprise the patient's location of residence, duration of gestation, as well as the sex and race of the infant.

For the collection of data, it was subjected to analysis using the Microsoft Excel software to facilitate the organization of the data into suitable tables for further statistical analysis. The data set utilized encompassed both absolute numerical values and their associated relative percentages. The analysis involved an investigation of the variables and their distribution throughout the years under consideration. The absence of a necessity to submit an assessment report to the Research Ethics Committee can be attributed to the utilization of publicly available data in the study.

The statistical analysis was conducted using the GraphPad Prism 10 software, developed by GraphPad Software Inc., San Diego, CA, USA. The incidence data were subjected to analysis using simple linear regression to determine the inclinations, standard error, R^2^, and p-value. The analysis of variance (ANOVA) test was utilized to examine variance. A significance level (p) of 0.05 was used to define the 95% confidence interval for each trend. The determination of statistical significance was based on a p below the threshold of 0.05.

The research was conducted systematically, encompassing many discrete stages. The phases encompassed a comprehensive examination of pertinent scholarly literature, the execution of research activities, and the compilation of data specifically pertaining to the prevalence of congenital glaucoma in Brazil between 2017 and 2021. The findings derived from the research were later displayed and discussed.

## RESULTS

Congenital glaucoma is a multifaceted condition that is defined by an increase in IOP and is linked to a specific form of trabecular dysgenesis. Irreversible blindness represents the most severe prognosis, underscoring the importance of understanding the risk and epidemiological factors associated with this condition. Such knowledge is crucial for enhancing the understanding of this disease within the context of differential diagnoses of eye symptoms, facilitating timely treatment, and minimizing morbidity^2,3^.

The analysis of data sets obtained from the DATASUS-TABNET system enabled a thorough examination of the prevalence and characteristics of congenital glaucoma in Brazil from 2017 to 2021. The acquisition of pertinent data facilitated the development of an epidemiological profile in relation to the specified theoretical framework.

**Figure 1:**
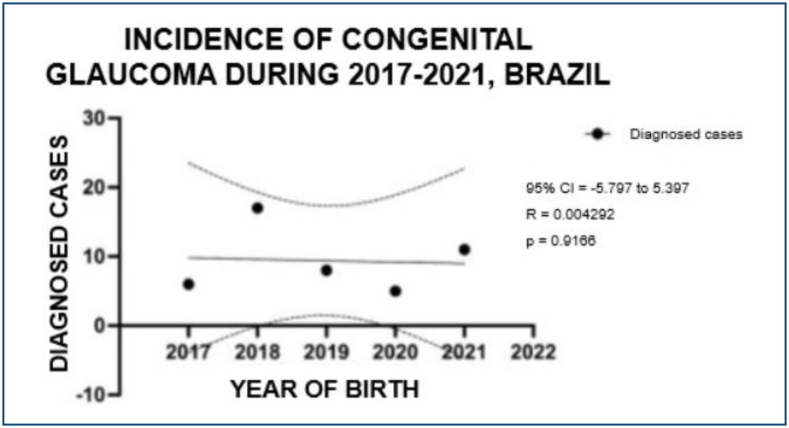
Analysis of the distribution of cases diagnosed with congenital glaucoma during the period 2017–2021, Brazil. Source: The authors.

During the study period, a total of 47 incidences of congenital glaucoma were found in Brazil, as shown in Figure 1. The distribution of occurrences throughout different years is as follows: in 2017, there were six cases, accounting for 12.77% of the total; in 2018, there were 17 cases, representing 36.17%; in 2019, there were eight cases, accounting for 17.02%; in 2020, there were five cases, representing 10.64%; and in 2021, there were 11 cases, accounting for 23.40%.

The analysis of the distribution of cases by Federation Unit (state), as demonstrated in Table 1, indicated that the states with the highest incidence of congenital glaucoma in the period evaluated were São Paulo (n=23, 48.94%), followed by Rio Grande do Sul (n=5, 10.64%) and Pernambuco (n=5, 10.64%). There were two notifications in each of the following states: Amazonas, Ceará, Minas Gerais, and Goiás. The states of Maranhão, Piauí, Alagoas, Sergipe, Rio de Janeiro, and Santa Catarina appointed one notification each.

**Table 1:** Demographic analysis of the infant diagnosed with congenital glaucoma, 2017–2021, Brazil.

Variants		n	%	95%CI	p-value
Infant's state of birth					
	Amazonas	2	4.26%	-0.2801 to 1.080	0.1942
	Maranhão	1	2.13%	-0.3553 to 0.7553	0.1604
	Piauí	1	2.13%	-0.3553 to 0.7553	0.1178
	Ceará	2	4.26%	-0.2801 to 1.080	0.1192
	Pernambuco	5	10.64%	-1.776 to 3.776	0.3505
	Alagoas	1	2.13%	-0.3553 to 0.7553	0.1178
	Sergipe	1	2.13%	-0.3553 to 0.7553	0.1178
	Minas Gerais	2	4.26%	-0.2801 to 1.080	0.1487
	Rio de Janeiro	1	2.13%	-0.3553 to 0.7553	0.1178
	São Paulo	23	48.94%	0.8134 to 8.387	0.0959
	Santa Catarina	1	2.13%	-0.3553 to 0.7553	0.1460
	Rio Grande do Sul	5	10.64%	-0.2417 to 2.242	0.1618
	Goiás	2	4.26%	-0.2801 to 1.080	0.1192
	Total	47	100.00%		

Source: the authors.

The rate of consanguinity is a contributing element that might lead to a rise in the number of instances within a specific geographical area. In populations characterized by a high prevalence index, the occurrence of primary congenital glaucoma may be significantly elevated, up to 10-fold, and the onset of the illness may occur at an earlier age and exhibit more severe manifestations^17^.

In relation to the circumstances surrounding pregnancy, it has been reported that all pregnant individuals had prenatal care during their pregnancies. However, it should be noted that prenatal visits do not encompass any targeted preventative measures for the occurrence of congenital glaucoma. Nevertheless, it is recommended to conduct genetic surveillance after the birth of a child with the aforementioned condition to evaluate the risk associated with future pregnancies^2,4,18^.

Significant disparities are observed between the genders of patients diagnosed with congenital glaucoma, as indicated in Table 2, in terms of underlying patient features. Around 60% of the cases (n=28) were found in male individuals, whereas the remaining 19 cases were found in females. The provided evidence supports the theory that the condition has a higher prevalence in males compared with females, with the underlying mechanism remaining unknown^9,17^.

**Table 2:** Epidemiological profile analysis of newborns diagnosed with congenital glaucoma, 2017–2021, Brazil.

Variants		n	%	95%CI	p-value
Infant's gender					
	Male	28	59.57	5.181–8.419	0.0397
	Female	19	40.43	0.7169–4.483	0.0007
	Male vs. female	47	100.00	3.487–4.913	<0.0001
Infant's ethnicity					
	White	23	48.94	0.4261–8.774	0.0482
	Black	1	2.13	-0.3553 to 0.7553	0.0550
	Mixed	22	46.81	1.162–7.638	0.1403
	Indigenous	1	2.13	-0.3553 to 0.7553	0.0609
Infant's birth period of gestation					
	28–31 weeks	3	6.38	-0.5106 to 1.711	0.0332
	32–36 weeks	8	17.02	0.1843–3.016	0.045
	37–41 weeks	36	76.60	2.604–11.80	0.1882

Source: the authors.

The analysis of the ethnic background of individuals diagnosed with congenital glaucoma revealed a notably greater prevalence among whites (n=23, 48.94%), which was statistically significant. Additionally, mixed individuals (n=22, 46.81%) also exhibited an elevated incidence of this condition. The number of notifications submitted by blacks and indigenous infants was limited to one each. The racial composition of the patients aligns with previous research on the epidemiological profile of this particular condition, where 83.3% of the patients were identified as individuals of mixed racial background^6^. In a study conducted in the United States, evaluating the Dallas Glaucoma Registry (DGR), assessing the incidence of congenital primary glaucoma across different ethnic groups revealed that Hispanics had a prevalence rate of 39%, Caucasians had a prevalence rate of 30%, and African Americans had a prevalence rate of 10%. The aforementioned indicators indicate that the breed of a subject has a significant impact on the probability of illness development^18,19^.

The duration of gestation is a crucial factor to consider when examining the epidemiological profile of congenital glaucoma. This pathological condition has been observed to potentially exhibit an association with prematurity, particularly due to the early development of internal angle glaucoma paralysis during a certain period in the third trimester of pregnancy^20^. In contrast to these findings, notable statistical disparities were detected across neonates of varying gestational durations. The study observed a subset of infants born from pregnancies lasting between 28 and 31 weeks (n=3, 6.38%) and 32 and 36 weeks (n=8, 17.02%) with a statistically significant association between these gestational age groups and the occurrence of congenital glaucoma. However, the group of infants born between 37 and 41 weeks (n=36, 76.60%) also exhibited an important correlation with the development of congenital glaucoma. This finding is supported by the data presented in Table 2. It is noteworthy to mention that there were no cases of congenital glaucoma observed in pregnancies occurring before 28 weeks gestation throughout the timeframe under investigation.

## CONCLUSION

Understanding the physiopathological basis causing congenital glaucoma remains an emerging topic, with research ongoing to unravel the processes of the condition. Therefore, in addition to the foregoing research, the epidemiological examination plays a crucial role in identifying the patient profile, addressing related risk factors, and allowing the identification of demographic and clinical trends.

Consequently, congenital glaucoma represents an exciting area of medical and scientific study, which demands interdisciplinary collaboration and continued research to improve our understanding, diagnosis, and treatment of this complicated disorder.
